# Inspirational Leadership and Innovative Communication in Sustainable Organizations: A Mediating Role of Mutual Trust

**DOI:** 10.3389/fpsyg.2022.846128

**Published:** 2022-07-28

**Authors:** Muhammad Toseef, Alina Kiran, Sufan Zhuo, Mahad Jahangir, Sidra Riaz, Zong Wei, Tauqir Ahmad Ghauri, Irfan Ullah, Suraya Binti Ahmad

**Affiliations:** ^1^Faculty of Management and Economics, Kunming University of Science and Technology, Kunming, China; ^2^Department of Management Sciences, University College of Zhob, Balochistan University of Information Technology, Engineering & Management Sciences (BUITEMS), Zhob, Pakistan; ^3^Faculty of Technology Management and Technopreneurship, Universiti Teknikal Malaysia, Malacca, Malaysia; ^4^Business School, University of International Business and Economics, Beijing, China; ^5^School of Business Administration, University of Lakki Marwat, Lakki Marwat, Pakistan; ^6^School of Governance and Public Policy, National University of Modern Languages, Islamabad, Pakistan; ^7^School of Ethnology and Sociology, Minzu University of China, Beijing, China; ^8^South Asian Studies Center Institute of Area Studies, Honghe University, Mengzi, China; ^9^School of Business Administration, Islamia University of Bahawalpur, Bahawalpur, Pakistan; ^10^School of Management and Economics, Beijing Institute of Technology, Beijing, China

**Keywords:** inspirational leadership, innovative communication, mutual trust, employee engagement, employee commitment, sustainable organizations

## Abstract

The possibility of accomplishing sustainable objectives is largely connected to the management and flourishing of an organizational system which keeps human capital engaged and committed. Our study investigated the association of inspirational leadership and innovative communication with employee engagement and commitment under the lens of leader member exchange theory. Specifically, we emphasized the mediating role of mutual trust in connection to social sustainability facets. A survey of data from employees in the manufacturing sector of Yunnan, China was utilized to test the hypothesized model. The study findings reported a significant association and came to the conclusion that a leader’s inspirational behavior coupled with innovative communication is a significant predictor of engagement and commitment in socially sustainable organizations. Moreover, mutual trust significantly mediated the relationship of innovative communication and inspirational leadership with employee engagement and commitment reaching the social perspective of sustainability. The current study added to the literature of sustainable organization by pointing out the social dimensions of sustainability.

## Introduction

Modern organizational phenomenon has premeditated a broader canvas of safer future generation along with attainable objectives. Organizational sustainability attracted academicians and entrepreneurs to develop such a system to change working habits within an organizational domain for the attainment of organizational goals and ultimate sustainability (Yu et al., 2018). The tri-dimension principle of sustainability has reached significance in literature, out of which the social dimension of an organization demands in-depth academic investigation compared to the economic and environmental dimensions. The social dimension is more variable as compared to the other two dimensions as it directly involves the behavior of society and behavior itself contains too much variance (Eizenberg and Jabareen, 2017). Workforce characteristics are reported to be elemental building blocks to sustainable organizations by the predicates of employee engagement and commitment in a single assortment (Di Fabio, 2017). The studies of Sehnem et al. (2019) elucidate that companies are molding their current business models toward the human sustainability perspective of the organization. Both academicians and managers agree on the fact that humans are the building blocks of an organization. If an organization wants to become more sustainable it must have to think about the sustainability of their employees as it will ultimately help in the achievement of organizational sustainability. Studies portrayed employee engagement as a multifaceted characteristic of socially sustained organization as it is witnessed by many academicians that engagement of employees helps in accomplishment of organizational objectives in good time and if the objectives are achieved regularly then social sustainability becomes a benefit for organizations (Chughtai et al., 2015; Mone and London, 2018). The pioneer of employee engagement Kahn (1990) elaborates that availability, psychological safety, and meaningfulness are crucial to organizations. Work mindset, dedication, absorption and employee commitment are characterized by a sustainable workplace (Schaufeli and Bakker, 2004). China is the biggest production operator in the world, which emphasizes correcting massive human liability and deficient employee engagement and commitment in the workplace to ensure a sustainable organization. The manufacturing sector is in serious need of adapting to and promulgating sustainable development (Dal Mas, 2019). Therefore, a greater question arises about sustainable organization beneath the perspective of employee engagement and commitment. It is therefore argued that employees’ level of commitment and their engagement in an organization may be the benchmark for sustainable organizations. To determine the diverse perspective of organizational sustainability, leadership is an optimistic path alongside dominant communication roots. Leadership is a trustworthy discipline to sustainable development and future generations of attainable goals (Slimane, 2012) because leaders in any organization support the engagement and commitment level of employees for the achievement of organizational goals and in turn sustainable development. Symbolically, communication shapes an individual’s future actions in building perceptions and the knowledge pool for accomplishing organizational objectives. Initially, the organizational perspective of communication was oversimplified to dissemination of information and later changed into innovation of communication (Juholin et al., 2015; Khan et al., 2019). This calls for organizational connectivity, leadership, process, structures, engagement, and commitment toward sustainable organization.

**FIGURE 1:**
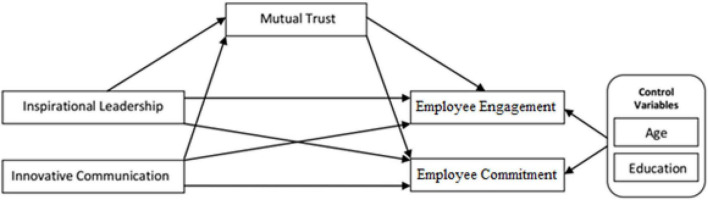
Conceptual model.

Previously, studies reported an inter-correlation between psychological capabilities and sustainable superior performance (Shamir and Lapidot, 2003; Lewicki et al., 2006; Carasco-Saul et al., 2015). There is also significant evidence of trust leading to increased abilities, satisfaction, commitment, and performance within an organization (Dirks and Ferrin, 2002, Costa, 2003). Furthermore, the social domain of sustainability includes the role of ethical leadership, employee engagement, trust, and self-efficacy along with moderating and mediating roles (Xu and Cooper Thomas, 2011; Mo and Shi, 2017; Park et al., 2018). Our study focused on innovative communication and the inspirational leadership relationship with employee commitment and engagement perspectives of a sustainable organization by investigating the mediating role of mutual trust (see Figure 1).

## Theoretical Perspective

### Sustainable Organization

The work of Brundtland and Khalid (1987) gave rise to the concept of sustainability and ever since academicians and industrialists have kept an eye on standards of performance. New challenges emerged that opened the gate for organizations to restructure operational standards (Higgins and Coffey, 2016). Environmental and social domains attained comparative interest in sustainable performance rather than economic performance (Chouinard et al., 2011). For the World Commission on Environment and Development [WCED] and Brundtland (1987), “development that fulfils current requirements without affecting future generations” is sustainability. Triangular principles of sustainability emphasize social, economic, and environmental concerns. Sustainable organization refers to implementing techniques that safeguard, sustain, and improve resources for future generations (Labuschagne et al., 2005; Goyal et al., 2013). Tri-pillar sustainable organizations safeguard the human system within inter-correlation and a supportive edge, Yusliza et al. (2020) also connected personnel procedures with the tri-pillar sustainability. They displayed a clear understanding of sustainability as an instrument for social and economic harmony. Work is vital to economic and environmental sustainability that regresses societal principles concealed in literature (Huq and Stevenson, 2018). The economic dimension works around materialistic benefits and financial gains, the environmental component assesses losses to the ecological system, while social sustainability is connected to the wellbeing of the civic circle, stakeholders, and workforce (Borrell, 2000; Gardberg and Fombrun, 2006). Studies have incorporated sustainability in supply chain, innovation, operational management, IT, and diverse business perspectives (Magon et al., 2018; Mavi and Standing, 2018; Danese et al., 2019; Inigo and Albareda, 2019).

Social sustainable organization is a quality human system which purely emphasizes fairness, justice, equity, and engagement. It is the way to balance social development with economic and environmental components of organization (Harris, 2003; Aggerholm et al., 2011). Organizations are liable toward society to uplift health, education, reduce poverty, and gratify employees along with economic growth (Haugh and Talwar, 2010; Closs et al., 2011). In order to serve society, entrepreneurs make investments to put forward a message of loyalty in return of services and social devotion (Golicic and Smith, 2013; Singh, 2018). According to Mani et al. (2018), labor practices and engagement are the integral pillar of social sustainability which remain a major organizational concern to connect communities. Moreover, commitmentand engagement injects a sense of sustainable action that influences every aspect of the organization and employee work life (Schaufeli et al., 2002; Banerjee et al., 2003; Jabbour and Santos, 2008; Schaufeli, 2013). The work of Ehnert (2009) reported self-knowledge, value responsiveness, reflection, thinking, and collaboration as sustainability contributing factors under an individual’s jurisdiction. These factors encourage an organization environment and sustain enduring work life. Employee-oriented packages of training, motivation, commitment, and engagement are operational and psychological maturity activities intended to ensure social sustainability in corporate sphere (Jerez-Gómez et al., 2007; Jackson et al., 2011; Manteklow, 2011). Our study promulgates leader member exchange theory in regard to our hypnotical relationship. The quality exchange relationship is being characterized by leader member exchange theory (LMX) to understand subordinate work behaviors. LMX explains the dynamic leader-subordinate interaction proposed by Scandura and Graen (1984), Graen et al. (1986), and Graen and Scandura (1987) over two approaches. Dyadic leadership theory detects trust and respect which binds an interactional relationship leaning on employee wellbeing and effectiveness (Erdogan and Bauer, 2015). Furthermore, quality relationships open up the role of LMX in generating performance and psychological fulfillment. LMX discovered social comparative status explaining variation, distribution, and behavioral characteristics of the members (Pearson, 2016).

### Inspirational Leadership and Sustainable Organization

Leaders pose charismatic abilities to inspire followers and attain desirable employees at the top of their operational expertise. These behaviors are time-effective across diverse cultures (Bass and Stogdill, 1990; Avolio and Bass, 2004). Leaders have evolved as strong navigators and lead through a complex market environment. Leadership is an explanation to implement sustainability using abilities like interpreting, predicting, and engaging teams and intellectuals in regards to a complex changing work environment (Mtcalf and Benn, 2012). Society is affectionate of sustainable benefits, and leadership is the central point of sustainable business, breakthroughs, and society. However, Slimane (2012) is of the view that leadership and social sustainability are dynamic organizational textures (Inness et al., 2010). Moreover, environmental, economic, and social adaptability characterize distinguishing leaders and their sustainable vision. Transformational style is collective in terms of corporate sustainability. A definitive achievement of a leader is to influence followers to do apparently unimaginable accomplishments. For sure, optimistic leaders motivate their followers to achieve undertakings and goals well past their own desires (Mtcalf and Benn, 2012).

Inspired leaders inspire, personalize, and stimulate intellect (Rabiul and Yean, 2021). Dionne et al. (2004) defines idealized influence as a leader’s capacity to convey a vision and/or demanding objective to subordinates while winning their confidence and commitment. Transformative leadership helps workers accomplish their goals by giving them specific attention (Nübold et al., 2013; Blomme et al., 2015). Employees are inspired by leaders’ intellectual stimulation to evaluate difficulties (Burns, 1978; Kark et al., 2003). According to Inness et al. (2010) employees who trust their leader are more likely to put in extra effort. Transformational leadership has been linked to work engagement in a recent study (Amor et al., 2020), and new empirical evidence backs this up (e.g., Chua and Ayoko, 2021). Employees who believe their leaders care about their professional development may have a better sense of purpose at work. If their leaders care about them, their workers should be able to handle the psychological demands of their employment (Schaufeli and Salanova, 2007). Insights into transformative leadership and employee engagement from several studies show that transformational leadership boosts employee morale (Bui et al., 2017). In a new practice, leadership style and atmosphere impact employee perceptions, devotion, involvement, and engagement (Appelbaum et al., 2015; Winasis et al., 2021). While Jeong et al. (2016) and Schmitt et al. (2016) claim that transformational leaders are more likely to engage employees who encourage strong communication, creativity, job engagement, and flexibility.

Employees and their leaders share a psychological bond. Transformative leaders in the workplace are intelligent, likeable, and proactive (Barker, 2002; Li et al., 2005; Babcock-Roberson and Strickland, 2010). Favorable working conditions foster emotional dependency and so affect commitment (Baruch, 1998; Baruch and Rousseau, 2019). In this manner, followers are emotionally connected and committed toward their job duties (Wang and Guan, 2018). According to Eisenbeiss et al. (2008), such thinking fosters an innovative and creative culture. A leader’s zeal, transparency, desire, and creativity may invigorate and inspire subordinates (Dai et al., 2020). Transformational leaders inspire and encourage their employees to work with pride and dignity (Khurosani, 2018). Employees feel more connected to a firm when their needs and expectations are satisfied. With the company’s strategic goals in mind, workers are ready to contribute toward organizational sustainability (Park et al., 2018; Bakri and Abbas, 2020). Further, Begun and Jiang (2020) argue that sustainable organizational culture is critical for increasing organizational productivity and providing businesses with a competitive strategic direction. It is critical for organizations headed by transactional leadership styles to be able to continually innovate. Organizational learning was found to have an indirect impact on the link between transformative leadership and long-term organizational sustainability. Thus,

H1a: Inspirational leadership behavior is positively associated with employee engagement.
H1b: Inspirational leadership behavior is positively associated with employee commitment.

### Innovative Communication and Sustainable Organization

Innovative communication is defined as the transmission of information across the stakeholders for better coordination and guidance to achieve the goal of innovative activities (Pearson, 2016). The innovative communication approach is an operational approach to organizational communication, improving trust and performance. Pearson stated that there should be a proactive communication strategy along with management’s role in information transmission to succeed in a business and social setup. Similarly, Tuckman’s (1965) group development model converged trust, cooperation, and commitment as a group which ended in efficiency and enduring performance. The upshot of a case study in Brazil highlighted a firm’s penetrating sustainability and need to underpin a communication system that fills the space between HR practices and suitable values. Kalla (2005) explained employee communication as a “social interaction through messages” organization pillar. Welch and Jackson (2007) affirmed that a system of good communication is associated with management’s ability to match decisions with stakeholder preferences. Communication and a participative environment within an organization lead to employee bonding and towards trust and employee engagement (Anderson and West, 2002; Vezzoli et al., 2012). And Snyder (1981) elaborated that when an organization strategy has the element of professionalism, respect, and discussion of differences and similarities, trust ultimately develops among members to share individual competencies into group strengths. The study of Diana (2014) used a balance scorecard approach for dissemination of internal-external information as a toolkit of sustainable performance proposed by Kaplan and Norton (1996). Pearson (2016) argued that innovative higher educational institutes are the backbone of social enterprises, entrepreneurial activities, and engagement with committed staff, sharing and connecting innovative ideas, and problem solving. Chidiebere et al. (2015) concluded that effective communication between the administrative and non-administrative staff serves as a performance management tool. Moreover, management should create an effective channel of communication across the organization so all stakeholders can offer feedback. Siti Nabiha (2019) wrote about the system of motivational practices as a seed to a tree that grows up with multiple HR actions toward sustainable organization. Moreover, sustainability principles embody enduring social wellbeing of the workforce drawn from the organization HRM system (Taylor et al., 2012). Hence, we proposed that:

H2a: Innovative communication is positively associated with employee engagement.
H2b: Innovative communication is positively associated with employee commitment.

### Meditation of Mutual Trust

Trust is an interpersonal marvel in light of connections between an individual and someone else or group of persons (Costa, 2003; Tzafir, 2004). Trust is additionally observed as a declaration of trust in organizations, which prompts agreeable behavior among individuals and groups inside and between associations (Nandhakumar and Baskerville, 2006). Organizational culture that promotes employee discussion surely provides a foundation to achieve synergy among the members (Edmondson, 1999). Welch and Jackson (2007) Tuchman’s team deployment model started with the need of a leader, shaping a system of effective communication. This necessitated a workforce surrounding the role, rules, direction, and supervision received. Trust is measured at the interpersonal level, mirroring the connection among employer, employee and, organization (Marlow and Patton, 2002; Jiang and Luo, 2018). An empirical study by Yue et al. (2019) explained the association of inspirational leadership and communication in connection to the meditational effect of organization trust. Their empirical work spotted trust as a mediator neighboring transformational leadership, job satisfaction, communication, and employee performance (Dirks and Ferrin, 2002; Shockley-Zalabak and Ellis, 2006; Braun et al., 2013).

A foremost association of trust and collaboration was found within an organization. Since breaching trust breeds distrust, keeping up trust requires cautious consideration from management. It gives the idea that organization leaders must trust dealings with followers (Brower et al., 2000; Reihaneh et al., 2010). Trust is influenced by levels of leadership connections, organizational viability, and communication (Tschannen-Moran, 2001; Yang and Lim, 2009; Johnson et al., 2012; Kang and Sung, 2017). Work connections described by trust may fortify participation, decrease clashes, increment organizational commitment, and reduce the propensity to leave (Costa, 2003; Kim and Brymer, 2011). Managers necessitate trust in expertise and commitment to workers, while welcoming their interest in the basic leadership process (Whitener et al., 1998). Trust in leadership, also conceptualized as “trust in management,” has been related to positive organizational results, including the aim for job satisfaction, turnover, and satisfaction with investment in basic leadership, overall execution, organizational engagement, and commitment (Kiffin-Petersen and Cordery, 2003; Dirks and Skarlicki, 2004; Lchner, 2013; Men and Tsai, 2016). Managers could use trust to obtain commitment and decision support. Additionally, behavioral loyalty and commitment to work create an environment that urges managers to develop trust in representative confidence (Whitener et al., 1998; Salanova and Schaufeli, 2008).

Trust is seemingly a developmental component of behavior in a way to combine feelings, attitude, and actions. It has worked as a psychological response inside and outside of a team (Fukuyama, 1996; Costa, 2003). The study of Erdem and Ozen (2003) concluded by quantitative analysis that affective and cognitive trust within employee-linked emotion was associated with group objectives. Communication is a vital contribution for the processing development of social relations among members. Communication is a crucial component of effective employee building (Holmes, 2012). The work of Hakanen and Soudunsaari (2012) supported these arguments by explaining that there is a group of factors but the chief component depends on communication toward the trust building process in getting high performance. Cooperation and solidarity is the best road map within an organizational climate. On the other hand, diversified psychological abilities like commitment with overall objectives and channels of communication constitute high achievement teams (Cheruvelil et al., 2014). Moreover, the best indirect channel to attain employee commitment includes a number of factors likes trust, cooperation, social interaction, and many more. Long-lasting psychological trust encourages commitment and engagement within the whole working environment (Roberts and Davenport, 2002; Macey and Schneider, 2008). Shared inner feeling for communication, trust, and commitment generates high-performing teams. Numerous studies pointed to the indirect effect of trust on high performance teams (Millward, 2009). Smith (1991) wrote about future employee communication. He pinpointed that the key will be the amount of respect and trust among the members and higher-ups. The concept of virtual teams is totally based on telecommunication using technological tools of information transmission (Powell et al., 2004). In the recent global village, telecommunication is the best way to ensure mutual trust and resultant teamwork (Kanawattanachai and Yoo, 2007). Transformational behaviors exhibit ethical performance of leaders which reflects desirability of justice and morality-inspirable functions (Walumbwa et al., 2008). Social exchange theory argued that work specification is well known to both organizational parties as what to perform. Under inspirational leadership, employee performance is an outcome in exchange of gains (Cropanzano and Mitchell, 2005). The way organizational leaders tolerate communication, inspiration, and moral conduct develops an atmosphere of mutual trust in exchange for secure enduring performance. LMX theory explicates communication as a working variable for the interactional relationship of leadership with employees under “in-group” categorization showcasing communication and cooperation (Graen and Scandura, 1987). The scholars Dienesch and Liden (1986) argued that trust, loyalty, and respect feature in the working relationship. Furthermore, Gerstner and Day (1997) reported outcomes of the exchange relationship in job satisfaction, commitment, and high performance. Group dynamics are critical to LMX interactions and to this end, a leader’s behavioral perspective influences employee communication, efforts, and commitment (Maslyn and Uhl-Bien, 2005; Hu and Liden, 2013). LMX quality creates a job attitude of commitment, satisfaction, engagement, and desirable employee behavior that is what an organization is hoping to achieve and sustain (Dulebohn et al., 2012). The above arguments suggest the following hypotheses:

H3a: Mutual trust mediates the positive relationship between inspirational leadership and employee engagement.
H3b: Mutual trust mediates the positive relationship between inspirational leadership and employee commitment.
H4a: Mutual trust mediates the positive relationship between innovative communication and employee engagement.
H4b: Mutual trust mediates the positive relationship between innovative communication and employee commitment.

## Materials and Methods

### Participants

China is the leading economic partner in the world with a highly influential rate of products and services across the globe. The belt & road initiative opened up mega projects in Asia and outside in order to develop structural networks like roads, industrial zones, power sectors, housing, and many more. All of these circumstances mean that the manufacturing industry has to come up with an internal bonding and humanitarian philosophy to make the most of this opportunity and ensure an enduring future. This is only practical for behavioral, operational, and rational decisions from the leadership to achieve sustainable performance. This study included managerial staff working in the manufacturing industry of Yunnan, China for primary data collection based on the convenience sampling technique (Boakye, 2015). The researcher ensured complete compliance with ethical consideration. None of the respondents were forced to give personal details and their identification is not visible in this research. Hence the anonymity of the participants is being ensured.

We divided data collection into two parts. Independent variables were separated from dependent variables to avoid common bias (Podsakoff et al., 2012) and the mediator required two waves for the mediated path (Cole and Maxwell, 2003). First we collected information on IL and IC, secondly we gathered data on MT, EC, and EE from the same employees after a 1-month interval in a different department. Data confidentially and willingness was ensured, a response of 175 questionnaires was received. Eliminating missing responses, a final number of 152 questionnaires was used. In terms of age, 22% were 21–25 years, 41% were 26–30 years, 27% were 31–35 years, and 6% were 36-40 years, while 4% were above the age of 40 years with mean score: M = 2.27 and SD = 0.963. As for education, 22% had a higher secondary level certificate, 28% had a bachelor’s degree, 47% had a master’s degree, and 3% had above a master’s degree with M = 2.11 and SD = 0.77.

### Measures

This study interacted with respondents using an adopted survey instrument with a five-point scale. Five items were used to measure innovative communication (Liang et al., 2007). Mutual trust was measured by a five-item checklist of Johnson-George and Swap (1982) to assess the mediating effect. Employee engagement was determined by a five-item survey by Schaufeli et al. (2002). Employee commitment was assessed by a five-item questionnaire by Wolfeld (2010). Inspirational leadership was measured by a five-item survey by Avolio and Bass (2004). In the study, we controlled for demographics such as age and education level that had predictive power in relation to employee outcomes (Organ and Ryan, 1995).

### Data Analysis

We investigated the study model using partial least square (PLS-3), due to its multiple processing and handling errors in unobserved variables, separation from multivariate normal distribution, and strong theory prediction power (Gefen et al., 2000; Chin et al., 2003) for data analysis specifically SEM-PLS for testing study hypotheses. PLS is the best in terms of the bootstrapping re-sampling technique for estimation of t-values (Temme et al., 2006).

## Findings

### Measurement Model

A measurement model deals with the assessment of construct validity by applying convergent and discriminant validity and composite reliability. Convergent validity indicates whether items, measuring the same construct, have average variance extracted (AVE) over 0.5 (Fornell and Larcker, 1981) as well as confirmed factor loading over 0.60 and 0.7 (Gefen and Straub, 2005). All of the variables verified met the criteria of having AVEs (0.52 to 0.73) over 0.5 (Table 1). However, construct reliability (CR) verifies the internal consistency of the set of items. The values of CR (Table 1) ranged from 0.84 to 0.93 which were well above the 0.70 acceptable threshold (Bagozzi and Yi, 1988). Here the construct reliability was assured based on said results.

**TABLE 1:** Construct reliability and validity.

Constructs	Items	Loadings	CR	AVE
Innovative	IC1	0.81	0.852	0.536
communication	IC2	0.813		
	IC3	0.678		
	IC4	0.664		
	IC5	0.682		
Inspirational	lS1	0.682	0.861	0.554
leadership	lS2	0.699		
	lS3	0.811		
	lS4	0.736		
	lS5	0.785		
Mutual trust	MT1	0.704	0.848	0.529
	MT2	0.735		
	MT3	0.81		
	MT4	0.699		
	MT5	0.683		
Employee engagement	EE1	0.833	0.931	0.731
	EE2	0.88		
	EE3	0.844		
	EE4	0.882		
	EE5	0.834		
Employee commitment	EC1	0.819	0.856	0.599
	EC2	0.676		
	EC3	0.82		
	EC4	0.772		

## Results

The degree of construct differentiation by the items reported discriminant validity. The verification of discriminant validity was examined (Table 2) as the square root of the AVEs which was well above the inter-correlation between the constructs reported by Chin (1998) and Yi and Davis (2003).

**TABLE 2:** Construct inter-correlation.

	IC	IL	MT	TC	TC
IC	**0.732**				
IL	0.415	**0.744**			
MT	0.668	0.613	**0.727**		
EC	0.652	0.675	0.771	**0.774**	
EE	0.595	0.558	0.711	0.671	**0.855**

*The bold diagonal values represent the square root of AVEs. IC, innovative communication; IL, inspirational leadership; MT, mutual trust; EC, employee commitment; EE, employee engagement.*

### Structural Model

The extant of variance in endogenous variables by exogenous and estimation of path coefficient was assessed using a structural model. We examined the significant association of inspirational leadership (IL) and innovative communication (IC) with employee engagement (EE) and employee commitment (EC). The study results reported a significant association of inspirational leadership (β = 0.35, t = 4.51, *p* < 0.05) and innovative communication (β = 0.45, *t* = 4.87, *p* < 0.05) with employee engagement.

Similarly, for the second dependent variable, a significant association was found of inspirational leadership (β = 0.49, *t* = 7.91, *p* < 0.05) and innovative communication (β = 0.44, *t* = 5.81, *p* < 0.05) with employee commitment (Table 3), supporting H1a–H1b and H2a–H2b. The study results also reported an insignificant effect of age (β = −0.01, *t* = 0.18, *p* > 0.05; β = −0.11, *t* = 1.88, *p* > 0.05) and education (β = −0.00, *t* = 0.16, *p* > 0.05; β = −0.02, *t* = 0.50, *p* > 0.05), proving no confounding effect of both controlling variables regarding employee commitment and employee engagement. Both of the models represented R^2^ = 0.92 and = 0.48 for employee commitment (92%) variance and employee engagement (48%) variance, respectively.

**TABLE 3:** Total effect.

	Beta	SE	t	*p*
Age → employee commitment	–0.01	0.056	0.183	0.855
Age → employee engagement	–0.116	0.061	1.887	0.059
Education → employee commitment	0.008	0.047	0.164	0.87
Education → employee engagement	–0.029	0.058	0.503	0.615
Innovative communication → employee commitment	0.449	0.077	5.819	0.000
Innovative communication → employee engagement	0.459	0.094	4.874	0.000
Inspirational leadership → employee commitment	0.49	0.062	7.911	0.000
Inspirational leadership → employee engagement	0.358	0.079	4.518	0.000
				

### Mediation Testing

In order to investigate the mediation of mutual trust, we adopted the steps of Baron and Kenny (1986), Liang et al. (2007), Shao et al. (2016), and Ilyas et al. (2020). We first examined the significant effect of independent variables (IC and IL) on dependent variables (EC and EE) with no mediator. The study results reported a significant effect of innovative communication and inspirational leadership on dependent variables employee commitment and employee engagement, respectively (Table 3). Secondly, we examined the significant effect of independent variables (IC and IL) on the mediator (MT). The study results (Table 4) met this condition with innovative communication (β = 0.50, *t* = 6.71, *p* < 0.05) and inspirational leadership (β = 0.40, *t* = 7.12, *p* < 0.05) having a significant effect on the mediator mutual trust. Then a significant effect of the mediator (β = 0.41, *t* = 5.28, β = 0.45, *t* = 5.12, *p* < 0.05) was found for both dependent variables. Finally we examined the significant effect of independent variables (IC and IL) on dependent variables (EC and EE) controlling for the mediator (MT). We found a significant effect of innovative communication (β = 0.24, *t* = 3.15; β = 0.21, *t* = 2.47, *p* < 0.05) and inspirational leadership (β = 0.32, *t* = 5.77; β = 0.19, *t* = 2.33, *p* < 0.05) on dependent variables employee commitment and employee engagement, respectively (Table 4).

**TABLE 4:** Direct effect.

	β-value	Std. error	t-value	*p*-value
Innovative communication → mutual trust	0.501	0.075	6.712	0.000
Innovative communication → employee commitment	0.242	0.077	3.156	0.002
Innovative communication → employee engagement	0.212	0.086	2.47	0.014
Inspirational leadership → mutual trust	0.404	0.057	7.122	0.000
Inspirational leadership → employee commitment	0.323	0.056	5.776	0.000
Inspirational leadership → employee engagement	0.193	0.082	2.339	0.020
Mutual trust → employee commitment	0.411	0.078	5.286	0.000
Mutual trust → employee engagement	0.452	0.088	5.122	0.000

Moreover, this study employed bootstrapping: a non-parametric re-sampling technique to examine mediation using the significance of indirect effect proposed by Preacher et al. (2007). Here, the indirect effect was also significant for inspirational leadership (β = 0.16, *t* = 4.09, *p* < 0.05; β = 0.18, *t* = 4.64, *p* < 0.05) and innovative communication (β = 0.20, *t* = 3.83, *p* < 0.05; β = 0.22, *t* = 3.87, *p* < 0.05) on dependent variables employee commitment and employee engagement through mutual trust (Table 5). Here the results verified the mediation with a reduction effect, leading to partial mediation supporting H3a & H3b and H4a & H4b.

**TABLE 5:** Indirect effects using bootstrapping.

	β-value	Std. error	t-value	*p*-value	Remarks
Innovative communication → mutual trust → employee commitment	0.206	0.054	3.838	0.000	Mediation
Inspirational leadership → mutual trust → employee commitment	0.166	0.041	4.090	0.000	Mediation
Innovative communication → mutual trust → employee engagement	0.226	0.058	3.879	0.000	Mediation
Inspirational leadership → mutual trust → employee engagement	0.183	0.039	4.645	0.000	Mediation

## Discussion

An organization is an operational workplace for employees to join, learn, and utilize expertise to attain sustainable outcomes. Inspirational behavior and information dissemination by the leaders operationalize trust in the working environment. These cumulative components promote an enduring saga of employee engagement and commitment. A trustworthy environment enhances work psychology and management-subordinate collectivity. This study exposed the significant association between inspirational leadership and innovative communication with mutual trust, employee engagement, and commitment. The positive significance indicated that organizations’ interplay between communication and inspirational behavior like planning, organizing, etc. are instrumental approaches to psychological employee wellbeing to sustain the social organization domain.

Our study revealed that the association of inspirational leadership behavior and social sustainable performance were linked (Whetten and Cameron, 2011; Ilyas et al., 2020), verifying the recommendation of Beech and Crane (1999) as the pipeline of successful employee working, meaning that leaders that utilize support, direction, and the work platform to attain organizational achievements. Borell and Como (1999) suggest this by reporting employee failure in the absence of supportive leaders. A strong foundation of employee communication supplies workers with self-trust and trust of others as part of a joint venture (Hoegl and Gemuenden, 2001; Rajhans, 2012; Yue et al., 2019). Moreover, increasing the amount of information and feedback strengthens self and organizational trust. Our results found a positive association of mutual trust with employee engagement and commitment in line with the studies of Aquino and Reed (2002), Costa (2003), Khan et al. (2019), and Ilyas et al. (2020). They highlighted the fact that trust engenders behavioral origins and unfolds synergic power between employees. The study of Hakanen and Soudunsaari (2012) concluded the value of communication and trust as a building block of employee commitment. Moreover, trust grows through active communication, mutual respect, and shared experience. The positivity of results revealed that a sound system of effective communication ensures individual and organizational trust among the members to attain a feeling of team bonding.

Trust is a meditational factor in between the relationship of inspirational leadership, innovative communication, and social sustainability components as supported by Oreg (2006), Yue et al. (2019), and Ilyas et al. (2020). The findings elucidate prioritizing communication and trust in employee psychology by organizational leadership. Leaders that are known to be essential and trustworthy will retain the commitment, engagement, and work connectivity of their employees. Staff look to motivational support from leaders to encourage their intellectual and technical skills (Larsen et al., 1991). The findings of Lorraine Nelsey et al. (2012) verify the integral connection of leadership and employee services. Trust works as a second line of managerial authority to get work done from workers (Bijlsma and Koopman, 2003; Joseph and Winston, 2005; Herold et al., 2008; Sohmen, 2013), meaning that trust enlarges interpersonal potency between management and workers’ affiliation. Hence, this study suggests that organizational insight is important for enduring development and social sustainability.

## Conclusion

Our study investigated the relationship of inspirational leadership, innovative communication, and mutual trust with employee commitment and engagement in the manufacturing sector by applying a survey approach. The positive nature of results determined how to get the best out of HR capital in coping with organization goals and also securing an organization’s future by the humanistic work approach. Furthermore, we studied the meditational role of mutual trust in the relation between inspirational leadership and innovative communication with employee engagement and commitment. A structured equation modeling technique was used for testing our study model. Above all, the findings heavily elaborated the positive influence of inspirational leadership and innovative employee communication via mutual trust on employee engagement and commitment which shields sustainable performance. Management should put human wellbeing first to safeguard the work environment and ensure the attainment of organizational objectives.

### Practical Implication

This study contains several practical implications that have added to the research on organizational decision-making. First, the results supported our prediction that trust is a key component in the manufacturing sector to gaining managerial support by other stakeholders to import work quality. Secondly, our findings advocate the significance of managerial behavior and communication for sustainable performance. Thirdly, in the light of empirical findings, a trustworthy environment can be guaranteed to generate synergic power to assist management and obtain productive efforts. Finally, empirical findings revealed that every organization feels how important worker wellbeing is to the success of the company at a higher level. Our study strongly supports the management of industries to develop a sound system of communication and trust to motivate the workforce in connection with socially sustainable organizations.

### Theoretical Implications

Our study provides insight about social sustainability in many ways, especially in connection to the mediating role of mutual trust in the relationship between inspirational leadership and innovative employee communication with employee engagement and commitment as social dimensions of sustainability.

First, our study strengthens the coalescent part of inspirational behavior and innovative communication that ensures sustainability as a human capital preservation technique for the organization. Secondly, our findings clearly describe the positive role of trust that structures individual’s behavior, communication, and teamwork. Meaning that a trustworthy environment generates synergic power that obtains productive employee efforts. Secondly, our study expands the field of work by incorporating the mediating path that declares the best route to ensure employee engagement and commitment. Shared feelings of trust are indispensable by having clear knowledge of a vision and mission that helps employees to work as a team. The theoretical perspective of the Tuckman (1965) and Smith (1991) group development model supported the foundational study base. Their work explained that initially the members require information, directives, and leadership to understand their role in the light of policy to develop self-belief and trust, therefore boosting commitment and employee engagement. Finally, our study contributes to the literature by adopting a contextual approach to test the hypothesized model and validate it in the Chinese manufacturing sector to answer the human dimension of sustainability.

## Limitations and Future Direction

The study has some limitations like the sample size, which means that the findings are applicable to a limited extent but may not be appropriate to study sustainable performance over a wide area (Ilyas et al., 2020). Our study contributed by validating the scale in the study setting supported by the findings in particular. Previously, scholars found that communication and mutual trust are predictors of employee work, but our study determined that shielding trust was needed to achieve sustainable working in an industrial context. This study will provide a base for further study by assessing components of leaders’ behavior, trust, and performances, by extending academic research by investigating the meditational role of mutual trust in relation to leadership and social sustainable facets, and by adding further leadership dimensions under the model proposed by Bass (2000) to expand this theoretical hypothesis for in-depth empirical findings and a better organization perspective in the industrial context.

## Data Availability Statement

The original contributions presented in the study are included in the article/supplementary material, further inquiries can be directed to the corresponding authors.

## Ethics Statement

The studies involving human participants were reviewed and approved by Kunming University of Science and Technology. Written informed consent for participation was not required for this study in accordance with the national legislation and the institutional requirements.

## Author Contributions

MT, AK, and SZ data curation, formal analysis, and original draft of the manuscript. MJ, SR, and ZW contributed to the revision of the manuscript. IU and TG contributed to the writing, review, and editing of the manuscript. SA contributed to the supervision and guidelines.

## Conflict of Interest

The authors declare that the research was conducted in the absence of any commercial or financial relationships that could be construed as a potential conflict of interest.

## Publisher’s Note

All claims expressed in this article are solely those of the authors and do not necessarily represent those of their affiliated organizations, or those of the publisher, the editors and the reviewers. Any product that may be evaluated in this article, or claim that may be made by its manufacturer, is not guaranteed or endorsed by the publisher.
